# Carbohydrate-based biosensors for enhanced pathogen detection

**DOI:** 10.1007/s00216-025-06272-6

**Published:** 2025-12-26

**Authors:** Nada Elgiddawy, Hassan Mohamed El-Said Azzazy

**Affiliations:** 1 Department of Biotechnology and Life Sciences, Faculty of Postgraduate Studies for Advanced Sciences (PSAS), Beni-Suef University, Beni Suef, 62511 Egypt; 2 Department of Chemistry, School of Sciences & Engineering, The American University In Cairo, AUC Avenue, SSE # 1184, P.O. Box 74, 11835 New Cairo, Egypt

**Keywords:** Carbohydrate-based biosensors, Carbohydrate-pathogen interaction, Multivalent glycoconjugate, Carbohydrate functionalized surfaces, Pathogen detection

## Abstract

Carbohydrate-based biosensors represent a cutting-edge class of bioinspired diagnostic platforms that exploit the inherent specificity of glycan–protein interactions for pathogen detection. Carbohydrate-functionalized biosensing systems offer remarkable advantages in terms of sensitivity, selectivity, and biocompatibility, positioning them as compelling alternatives to conventional antibody- or nucleic acid-based assays. By mimicking natural recognition mechanisms, these interfaces enable rapid, scalable, and robust capture of microbial targets, even in complex biological matrices, thereby paving the way for detection platforms suitable for clinical diagnostics, environmental monitoring, and food safety applications. Recent advancements in glycan chemistry, nanotechnology, and surface functionalization, particularly the precise control of ligand density, orientation, and spatial arrangement, have significantly enhanced the performance of these biosensors. This review highlights the design principles, detection strategies, and emerging applications of carbohydrate-based biosensors targeting a broad spectrum of studied pathogens. It underscores their transformative potential in advancing point-of-care diagnostics and enhancing infectious disease surveillance.

## Introduction

Microorganisms are present everywhere in the environment and could significantly influence human health [1]. Infectious diseases remain a major contributor to global mortality, with the greatest impact seen particularly in developing countries [2]. Even though the incidence of infectious diseases and food poisoning caused by microorganisms has decreased over the years, the overall number of these diseases remains significant. Recent outbreaks of infectious diseases such as COVID-19 [3], Ebola, Zika, dengue, Middle East respiratory syndrome (MERS), severe acute respiratory syndrome (SARS), and influenza, along with the growing threat of antimicrobial resistance, have raised concerns about whether the current global health system is adequately equipped to effectively address the evolving spectrum of infectious diseases [4]. A key prerequisite for countering opportunistic and harmful pathogens is the development of sensitive, accurate, and rapid detection methods which are also crucial for medical diagnostics and national security [1, 5].

Conventional diagnostic techniques were used in clinical, industrial, and environmental settings, but they often lack cost-effectiveness, sensitivity, and specificity, particularly in complex samples. Microscopy and culturing are slow and prone to errors. Although PCR, ELISA, and flow cytometry provide high sensitivity, they demand trained personnel, expensive reagents, sophisticated laboratory facilities, and complex equipment that hinder portability [6, 7]. Additionally, pathogen enrichment is necessary to provide a sufficient number of cells for reliable detection, adding further delays [8]. Biosensors have emerged as a pioneering tool for pathogen detection by combining a selective biorecognition element (e.g., antibodies, nucleic acids, lectins, and carbohydrates) with a sensitive transducer to generate measurable signals for specific and quantitative identification [9, 10]. Several biosensors were developed to enable portable microbiological testing in various settings, including hospitals, field environments, and at the point of infection [11].

Researchers explored the potential of carbohydrate in preventing pathogen adhesion, utilizing carbohydrate-based nanomaterials, combating microbial infections, and developing carbohydrate-based vaccines [12]. Subsequently, carbohydrate-based recognition systems offer a potent alternative for pathogen detection by leveraging multivalent glycan–pathogen interactions to achieve high sensitivity and selectivity [13]. This review outlines key molecular interactions with bacteria, viruses, and toxins, and examines advanced immobilization techniques. It also evaluates the strengths, limitations, and emerging trends in carbohydrate-based biosensing, with emphasis on applications in diagnostics, food safety, and biosecurity.

## Biorecognition moieties

### Overview of biorecognition strategies

Biosensors for pathogen detection rely on biorecognition moieties, molecules that selectively interact with microbial targets. Based on their mechanism, biosensors are categorized into biocatalytic or affinity-based [14]. Biocatalytic sensors use enzymes or whole cells as markers to generate measurable signals, offering high sensitivity but requiring multistep reactions and controlled conditions, which limit their portability and rapid use. In contrast, most pathogen biosensors are affinity-based [15], relying on specific, non-catalytic binding between the analyte and immobilized recognition elements. These include antibodies, nucleic acids, aptamers, peptides, carbohydrates, and bacteriophages, each coupled to a transducer that converts the recognition event into a measurable signal [9].

### Affinity-based recognition moieties

Antibodies remain the most established recognition elements due to their high affinity and specificity toward target antigens. They enable rapid and selective pathogen detection and discriminate [16]. However, antibody-based biosensors often face batch variability, poor stability, and high costs, with binding performance sensitive to temperature, pH, and protein conformation [9]. Nucleic acids, particularly DNA and RNA oligonucleotides, serve as probes that hybridize with complementary sequences from pathogen genomes [17]. Their high specificity and easy synthesis make them attractive for rapid genetic detection. Nonetheless, nucleic acid-based sensors typically require additional steps for sample preparation and amplification to achieve practical sensitivity [2, 9]*.* Aptamers, short single-stranded nucleic acids that fold into diverse structures, can selectively bind proteins, small molecules, or even whole cells [18]. Aptasensors provide synthetic flexibility and reusability, yet their application in detecting whole bacteria is limited because aptamers often favor small molecules and a single aptamer may not effectively recognize heterogeneous bacterial surfaces, occasionally leading to false negatives [9]. Molecularly imprinted polymers (MIPs) offer a synthetic alternative to biological receptors, formed by creating molecular cavities complementary to the target [9, 19]. MIPs are inexpensive, robust, and reusable, but conventional imprinting methods often generate heterogeneous binding sites and face issues with template removal and cross-reactivity [9, 20]. Bacteriophages, viruses that infect specific bacteria, provide high selectivity with minimal cross-reactivity, and the ability to amplify signals through replication within the host improves sensitivity. They are cost-effective and highly specific but are limited by narrow host ranges, environmental sensitivity, and regulatory challenges in biosensor development [21, 22]. Carbohydrates are an emerging and powerful class of recognition elements in biosensor design. These systems effectively mimic biological recognition pathways for highly specific detection of whole cells and toxins [7]. Their structural tunability and chemical and thermal stability enable strong, selective, and reusable binding with superior stability and reproducibility compared to other biorecognition moieties. Thus, carbohydrate-functionalized biosensors present a robust and scalable platform for next-generation applications requiring high sensitivity, throughput, and long-term reliability [23, 24].

## Carbohydrate architecture and its role in pathogen recognition

### Structural diversity and synthesis of carbohydrates

Carbohydrate**s** represent a major class of biomolecules, built from monosaccharides like glucose, mannose, galactose, fucose, sialic acid, N-acetyl galactosamine, and N-acetylglucosamine, linked by glycosidic bonds formed via condensation reaction [25]. Due to their ability to form both linear and branched polymeric structures, carbohydrates exhibit vast structural diversity, making them a better information storage medium, compared to antibodies or DNA. Moreover, they are more stable, less prone to denaturation, and their smaller size allows for higher densities per unit surface area, enhancing binding affinity and sensitivity [26].

Despite significant progress, the synthesis of complex carbohydrates remains challenging, costly, and often yields limited quantities. Unlike peptides or nucleic acids, carbohydrates cannot be easily amplified, making them expensive and less accessible. Their structural complexity arising from variations in branching, linkage positions, anomeric configurations, and protecting group strategies further complicates their synthesis and application. Recent advances such as automated solid-phase, one-pot chemical, and chemoenzymatic approaches have improved efficiency, yet issues of enzyme availability, substrate cost, and scalability persist. Efforts to engineer microbial systems for enzyme expression offer promise, thereby expanding opportunities in biological research and biosensor development [12].

### Carbohydrate pathogen interaction

Carbohydrates are abundant in living organisms and play key roles in cellular processes. They encode complementary information for cellular functions, mediating sugar-based signals involved in communication, recognition, adhesion, and signaling, and pathogen entry referred to as the “glycan code.” They too serve as markers of disease progression, immune responses, and infection [23, 25]. Carbohydrates are crucial in host–pathogen interactions primarily through the glycocalyx—a dense carbohydrate-rich layer on cell surfaces that serves as a critical determinant of bacterial and viral pathogenesis [25, 27]. Pathogenic lectins and other carbohydrate-binding domains (e.g., toxins, adhesins) target glycans in the host cell glycocalyx exploiting this “glycolandscape” to initiate infection and enhance virulence [28, 29]. This sugar-specific adherence enables pathogens to invade, colonize, and release virulence factors. Without functional lectins, many pathogens fail to establish infection, highlighting their critical role in disease onset. Translating these adhesion mechanisms may reveal new strategies to prevent and treat infectious diseases [30, 31].

Bacterial adhesion, a key initial step in infection, often involves the binding of surface lectins to complementary carbohydrates on host tissues, facilitating colonization and subsequent biofilm formation. Many bacterial strains synthesize surface lectins, known as fimbriae or pili, which are typically submicroscopic, multi-subunit protein structures. *Escherichia coli*, a Gram-negative pathogen, is among the most extensively studied bacteria for its well-characterized glycan-binding pili, including mannose-specific type 1 pili, galabiose-specific P pili, and N-acetylglucosamine-specific pili. Type 1 pili, found in most *E. coli* strains, mediate adhesion to mannose receptors on host cells in the bladder epithelium, leading to cystitis [7]. Beyond adhesion, pathogens frequently exploit host cell glycosylation to facilitate toxin binding and internalization. A well-characterized example is the *Vibrio cholerae* AB5 toxin, in which five lectin subunits specifically bind GM1 gangliosides on intestinal epithelial cells, enabling the internalization of the enzymatic A subunit that constitutively activates host signaling pathways, ultimately driving ion efflux into the intestinal lumen [32]. Similarly, viruses exploit carbohydrate-binding domains to mediate host cell attachment and enter cells. For instance, influenza virus hemagglutinin, being the most extensively characterized example, binds the sialylated receptor NeuAcα2-6Galβ1-4GlcNAc on human epithelial cells, facilitating membrane fusion, viral entry, and replication [33].

## Carbohydrate-based biosensors

**Fig. 1:**
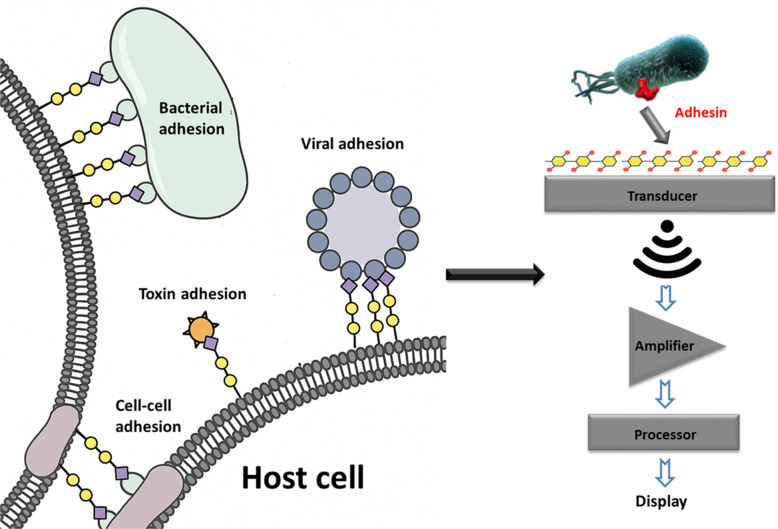
Natural multivalent interactions between pathogenic agents and carbohydrate ligands on host cell surfaces (left), and the development of carbohydrate-inspired biosensors (right)

Carbohydrates’ versatility supports the development of molecular fingerprints for diagnostics and allows for low-cost, high-throughput analyses. Their interactions with proteins such as enzymes, toxins, antibodies, and lectins enable broad specificity, allowing for the detection of diverse agents including bacteria and viruses on a single platform using only a few epitopes [25, 34]. Mimicking natural cellular interactions, carbohydrate-based biosensors support miniaturization and high throughput and require only picomole quantities of ligands (Fig. 1). Furthermore, they can detect pathogens in complex mixtures, and their nondestructive nature preserves sample integrity and allows for pathogen recovery and antibacterial susceptibility testing. Their broad interaction specificity allows microarray platforms that can identify diverse, and even novel, targets using a limited set of epitopes [35]. These unique features highlight their value in screening biological samples, detecting contaminants, and developing anti-adhesion therapeutics, underscoring their critical role in advancing pathogen detection technologies [17].

### Principles of carbohydrate-mediated recognition

Carbohydrates engage with lectins through a combination of hydrogen bonds, metal coordination, van der Waals forces, and hydrophobic interactions. The abundance of hydroxyl groups in carbohydrates creates complex hydrogen bond networks, while divalent cations such as Ca^2^⁺ and Mn^2^⁺ are essential, either by influencing the structure of the lectin binding site or by directly engaging the carbohydrate. In addition, hydrophobic interactions with specific sugar residues contribute to binding affinity and specificity. These complex interactions make it challenging to develop effective detection methods based on monovalent carbohydrate recognition [25].

Multivalent glycoconjugates, including glycopolymers and glycodendrimers, mimic natural recognition events and display greater avidity and specificity than monovalent ligands, enabling more precise cell receptor binding and improved analysis of cell interactions [25, 36]. For instance, glycopolymers containing mannose and an alkanethiol linker can be chemisorbed onto gold surfaces, where tuning the mannose to acrylamide ratio generates multivalent binding sites. Alternatively, cross-linked glycopolymers can be synthesized directly on gold, reducing polymer flexibility and conformational entropy [25]. Likewise, glyconanomaterials mimic natural cell surfaces by displaying carbohydrate functionalities that replicate the cell surface glycolipids and glycoproteins, thereby facilitating interactions with lectins, antibodies, and other carbohydrate-binding proteins [36, 37].

Effective nanoprobes that can detect, image, and profile microbes and cells will aid the understanding of the roles played by carbohydrates in the disease process and the development of new theranostic tools for disease prevention and treatment [38]. Techniques such as UV–Vis spectroscopy, fluorescence spectroscopy/microscopy, surface plasmon resonance (SPR), and quartz crystal microbalance (QCM) are commonly employed to study carbohydrate–protein interactions. Since carbohydrates poorly absorb light, optical methods often rely on fluorescence labeling, though this can introduce artifacts. This has driven the development of label-free biosensors, such as SPR and QCM, to more accurately study these interactions [25, 39].

### Immobilization and preparation strategies

The performance of carbohydrate-based biosensors relies on effective, reproducible, and stable surface immobilization that preserves the 3D structure, orientation, and accessibility of saccharide ligands for specific and reliable key–lock recognition. Carbohydrate is immobilized on substrates through three main strategies. Physisorption relies on weak interactions between carbohydrates and substrates such as nitrocellulose or polystyrene, often enhanced by attaching anchoring tails as lipid or polymer tails. Covalent immobilization involves constant chemical bonding, ensuring firm placement, controlled orientation, and preservation of their 3D structure for reliable biosensing. Techniques such as reductive amination, carbodiimide coupling, and click chemistry link glycans via functional groups, yielding stable and reproducible surface densities. Underivatized saccharides can be immobilized by activating the reducing end or hydroxyl groups to react with amines, hydrazides, or oximes, whereas derivatized saccharides bearing aglycons (e.g., thiols, disulfides) enable oriented, stable attachment through bifunctional linkers. Cross-linkers and coupling reagents also connect saccharides to surfaces with carboxyl, amine, or thiol groups. Although this approach provides stronger, more durable attachment than physisorption, it is limited by low yields, random reactivity, and incompatibility with non-reducing sugars. Bioaffinity-based methods utilize specific interactions, such as the streptavidin–biotin system or DNA-directed immobilization to anchor carbohydrates onto surfaces offering high stability, dense probe packing, and improved sensitivity compared to direct covalent methods, making them powerful tools for glycan and glycomimetic biosensor applications. Each method has its own advantages, particularly in terms of selectivity and maintaining desired carbohydrate structure under suitable storage conditions [25, 35].

## Applications in pathogen detection

### Carbohydrate-based assays

Several techniques have been developed to facilitate the rapid isolation and detection of pathogen-associated carbohydrate–binding interactions, as summarized in Table 1.

**Table 1:** Carbohydrate-based strategies applied in pathogen/toxin isolation, capture, detection, microarray screening, and receptor–ligand interaction studies

Carbohydrate (support)	Target	Assay principle	e
Fluorescein-labeled O-polysaccharides	Antibodies (in serum, whole blood and whole milk) against Gram-negative *Brucella *spp.,* Salmonella *spp.	Fluorescence polarization inhibition assays (FPIAs)	[40]
Mannose single-walled carbon nanotubes	*Anthrax* spores	Spores aggregation evaluated by CFU reduction assay	[41]
Biotinylated galabiose (Gal1,4Gal)	Zoonotic* Streptococcus suis*	Spore aggregation evaluated by a standard luminescence-based ATP	[42]
Glycan-coated magnetic nanoparticles (gMNP)	*Salmonella enterica* serovar Enteritidis	Isolation of pathogens from food confirmed byTEM	[43]
Dextrin coated magnetic and gold nanoparticles	*E. coli*	Extract and. detect from leafy green vegetables	[43]
Magnetic beads coated with various types of HBGAs	*Norovirus*	Magnetic Bead Capture Coupled with RT-qPCR	[44]
Biotinylated polysaccharides to avidin-coated microtiter wells	*Staphylococcus aureus* type 8* and Haemophilus influenzae* type b	ELISA	[45]
Oligomannose and a tetrasaccharide coated on gold nanoparticles	HIV and *Streptococcus pneumoniae*	ELISA	[46]
Glycan arrays made up of synthetic oligosaccharides 1 − 4, mimicking segments of the capping oligosaccharides found in leishmanial LPG	*Leishmania parasites*	Glycan Microarray Immunofluorescence Assay	[47]
Biotinylated trisaccharide (β-Man-(1 → 2)-α-Man-(1 → 2)-α-Man) onto a streptavidin-coated plate	*Candida species*	Glycoarray based ELISA	[48]
Manα1–2Rhaα1–2Gal (MRG)	*S. enterica sv. S. Paratyphi A*, *S. Typhimurium*,* S. Enteritidis*	Glycan Microarray Immunofluorescence Assay	[49]
Captured vesicles containing ganglioside GM1	Cholera toxin (CT)	SPR (receptor–ligand studies)	[50]
Ganglioside GM_1_ receptor analogs (SAM) on gold	CTx and *E. coli* heat-labile enterotoxin (LT)	QCM (capture toxin)	[51]

### Carbohydrate-based biosensors

In recent years, a variety of sensitive and cost-effective sensors and detection methods were introduced to help prevent outbreaks [43, 52] However, many of these methods require prior knowledge of the specific pathogen being analyzed. The diversity and extensive understanding of surface-displayed carbohydrates offer valuable insights for developing more universal detection methods, which are essential for early diagnosis during outbreaks and tailored to specific pathogens [23]. Table 2 provides a comprehensive overview of recently developed carbohydrate-based biosensors for pathogen detection. It highlights key characteristics such as the type of carbohydrate ligand, the targeted pathogen, detection method, transducer type, and sensitivity range. This summary demonstrates the versatility of carbohydrate recognition in biosensor design and the advancements in achieving high specificity and sensitivity for rapid and reliable pathogen identification.

**Table 2:** The characteristics of developed carbohydrate biosensors for pathogen/toxin detection

Target pathogen (viruses, bacteria/toxins)	developed biosensors	CHO moiety employed	Biosensors deign	detection strategy	minimal detection count	linear range	Reference
***Escherichia coli***	Fiber-optic biosensor	4-aminophenyl-α-D-mannopyranoside (AP-MAN)	AP-MAN receptor was covalently attached to LPFG	Optical spectrum analyzer (OSA)	10^3^ CFU/mL	10^3^–10^5^ CFU/mL	[53]
	Impedimetric biosensor	α-mannoside	Monolayer of α-mannoside self-assembled on a gold electrode	EIS	10^2^ CFU/mL	10^2^–10^3^ CFU/mL	[54]
	Label-free biosensor with two detection strategy	Mannose/Con A	Glycosylated polythiophene with fused quinone	Square wave voltammetry (SWV)	25 CFU/mL	1.0 × 10^2^–5.0 × 10^3^ CFU/mL	[55]
				QCM	50 CFU/mL	2.5 × 10^2^–2.5 × 10^8^ CFU/mL	
	Impedimetric biosensor	Mannose	Mannose-functionalized P3HT-b-P3TEGT nanoparticle	EIS	500 CFU/mL	10^3^–10⁷ CFU/mL	[56]
	Fluorescent biosensors	Glucose, stachyose, and raffinose	Glycosylated Cu:CdS quantum dots (QDs)	LDA	91.6% classification accuracy within 30 min	discrimination of Six bacterial strains	[57]
	Colorimetric biosensors	Mannose	monopod (MM) and tripod (MT) Mannose functionalized Gold nanoparticles (AuNPs)	Bacterial aggregation assay (solution color changes from red to blue)			[58]
	Optical biosensors	Mannose	Mannose-functionalized glycopolythiophenes	Visible absorption spectroscopy** (**unique red shifts)			[34]
	Colorimetric biosensors	Mannose	Mannose-derivatized PCDA	naked eye(color change from the blue to red) and can be measured using spectroscopy at 540 nm	**-**		[59]
	Fluorescent biosensors	Mannose	Multivalent mannose-functionalized fluorescent poly(p-phenylene ethynylene; PPE)	Brightly fluorescent aggregates of *E. coli* microscopically imaged	10^4^ bacteria		[60]
	Fluorescent biosensors (sensitive test strip)	Mannose	Electro spun polystyrene-co-maleic anhydride (PSMA) fibers, conjugated with mannose and tetraphenylethylene (TPE)	AIE Visual detection of bacteria under the illumination of a hand-held UV lamp	10^2^ CFU/mL	10^2^ to 10^5^ CFU/mL	[61]
	Fluorescent biosensors	Glucose	Glucose-based monoacrylamide and bisacrylamide	AIE for* E. coli* cell imaging and the detection	7.3 × 10^5^ CFU/mL	1.0 × 10⁶ to 1.7 × 10⁸ CFU/mL	[62]
	Fluorescent biosensors	Glucose	(AuNPs) stabilized with d-glucose-based bis(acrylamide)	Quenched and restoration of fluorescence upon interaction with Con A/*E. coli*	10^4^ CFU/mL		[63]
***Salmonella typhimurium***	Fluorescent-recoverable sensor	Glycopeptide-mimetic carbon dots (g-CDs)	(g-CDs) immobilized on Graphene oxide (GO) via 3-aminophenylboronic acid (3-APBA)	Fluorescence signal emitter	2370 CFU/mL	10^4^ to 10⁷ CFU/mL	[64]
***Salmonella enterica***	Optical biosensor	Mannose	B(OH)_2_**1**-mannan-functionalized droplets	Fluorescent double emulsion	< 10^4^ cells mL^–1^		[65]
***Helicobacter pylori***	Optical biosensor	Sialyl(a-2,3)lactose	Sialylglyco conjugates	Cuvette-based resonant mirror			[66]
	Optical biosensor	Lewis b tetrasaccharide		SPR	3000 CFU/mL		[67]
				DR LPG	10^2^ CFU/mL		
**Cholera toxin (CTx)**	Electrochemical biosensor	GM1	GM1-expressing Caco-2 cell membranes (CCM)	EIS	∼11.46 nM	100 ng/mL—1 mg/mL	[68]
	Colorimetric sensors	GM1	Ganglioside GM1-poly(diacetylene) liposomes	Visible absorption spectrum of blue/purple liposome solution	310 µg/mL		[69]
	Colorimetric bioassay	Lactose	Lactose-stabilized gold nanoparticles	Visible absorption spectrum	54 nM (3 µg/mL)		[70]
	Flow cytometry-based biosensor	GM1	Ganglioside GM1 on biomimetic phospholipid bilayers	FRET	below 10 pM		[71]
	Two-tiered FRET biosensor	GM1	Fluorophores (donor and acceptor) are covalently tagged to lyso-GM1	FRET	below 10 pM		[72]
**Shiga toxin (Stx)**	Mechanical biosensor	Gb3	Self-assembled monolayers (SAMs) of Gb3	QCM			[74]
	Chromatic biosensor	Gal-α1,4-Gal	Gal-α1,4-Gal glycopolydiacetylene (GPDA) nanoparticles	visible absorption	1200 units/µL	1200 to 7200 units/µL	[75]
	Optical biosensors	Gb3	Multivalent globotriose (P^k^ ligand)-functionalized gold nanoparticles (P^k^-AuNPs)	SPR			[76]
	Colorimetric biosensor	Gb3	globotriose@Au@FePor-TFPA-COP	Visible absorption			[77]
	Optical biosensors	Gb3	Silicon nitride (SiN) substrates with gold (Au) nanoparticles functionalization with a lactosyl derivative	RIfS	100 ng/mL	From (100 ng/mL to 1 µg/mL)	[78]
**SARS-CoV-2** spike glycoprotein	Colorimetric biosensors	HS	Heparan sulfate polysaccharide microarrays	GlycoGrip lateral flow assay	3.13 µg/mL	3.13 to 50 µg/mL	[81]
	Optical biosensors	N-acetyl neuraminic acid	Multivalent gold nanoparticles bearing N-acetyl neuraminic acid	paper-based lateral flow assay	5 µg/mL		[82]
	Optical biosensors	N-acetyl neuraminic acid-	N-acetyl neuraminic acid-functionalized, polymer [poly(hydroxy ethyl acrylamide)]-coated gold nanoparticles	Lateral flow assay			[79]
	Optical biosensors	2,3-Sialyllactose polymer-stabilized gold nanorods	2,3-Sialyllactose polymer-stabilized gold nanorods	Plasmonic bioassay	40 µg/mL spike protein		[83]
**Influenza Virus**	Colorimetric biosensors	Sialic acid	Sialic acid-polydiacetylene (PDA) liposome	Visible color change (blue/purple to pink/orange)	11 HAUs		[86]
	Colorimetric biosensors	Sialic acid	Water-soluble glycopolythiophenes with sialic acid	visible absorption spectrometry			[34]
	Optical biosensors	Sialic acid	Gold nanoparticles functionalized with a thiolated trivalent α2,6-thio-linked sialic acid derivative	Plasmonic bioassay/visible color change			[87]
	Colorimetric biosensors	SA receptors, with either α2,3- or α2,6-linkages	Glycan-functionalized gold nanoparticles (gGNPs)	Color change from red to purple and was measured spectrophotometrically			[88]
	Multiplex Microarray biosensor	α2,3 linked sialic acid moieties α2,6 linked sialic acid moieties	Immobilized on microarrays	AIR platform	0.06 µg/mL for lectins 9.29 × 10^5^ pfu/mL for human influenza and the avian-derived influenza		[24]
	Electrochemical biosensors	Two types of trisaccharides receptors terminating in sialic acid-α2,6-galactose and in sialic acid-α2,3-galactose)	Covalently attached to an amino-oxy-modified surface	FET	60 H5N1 or 6000 H1N1 proteins in a 20 µL sample		[89]
	Electrochemical biosensing	6′-Sialyllactose and 3′-sialyllactose on each gate area	Self-assembled monolayer by reagent, 3-aminooxypropyltriethoxy-silane (AOPTES)	Dual-channel integrated FET	10^0.5^ TCID_50_/mL	10^0.5^ to 10^8.5^ TCID_50_/mL	[90]
	Electrochemical biosensor	2,3-Sialyllactose	Immobilized on polycrystalline gold electrodes	Impedimetric			[91]
	Electrochemical biosensor	2,6-Sialyllactose	2,6-Sialyllactose functionalization of poly(ethylenedioxythiophene) (PEDOT)	OECT			[92]
	Mechanical biosensor	6′-Sialyllactose	6′-Sialyllactose immobilized gold electrodes	QCM	1/16 hemagglutinating units (HAU) virus		[93]
***Dengue* virus**	Electrochemical biosensor	Heparin	Heparin attached to the SWNT	Chemiresistive	1 virus		[95]

#### Bacteria

##### ***Escherichia coli***

*Escherichia coli* is a prevalent pathogen associated with numerous food- and waterborne infections, necessitating rapid and reliable detection strategies. Several label-free biosensors have been developed for *E. coli* detection, particularly exploiting mannose–bacteria interactions. A fiber-optic platform based on long-period fiber grating (LPFG) functionalized with 4-aminophenyl-α-D-mannopyranoside (AP-MAN) achieved reproducible performance but modest sensitivity (10^3^ CFU/mL) [53]. In contrast, electrochemical strategies demonstrated superior performance. For instance, self-assembled α-mannoside monolayers on gold electrodes enabled sensitive detection though with limited dynamic range (10^2^–10^3^ CFU/mL) restricting applicability for samples with highly variable bacterial loads [54]. Similarly, a glycosylated polythiophene with fused quinone enabled reagentless, label-free detection via dual pili–mannose and Con A mediated LPS-mannose interactions. This platform achieved remarkable sensitivity (25 CFU/mL by electrochemistry and 50 CFU/mL by QCM) with strong specificity [55]. More innovative approaches have exploited conductive carbohydrate-polymer interfaces. Mannose-functionalized P3HT-*b*-P3TEGT nanoparticle-based impedimetric biosensors achieved selective detection with a detection limit of 500 CFU/mL and a broad linear range of 10^3^–10⁷ CFU/mL [56]. These studies highlight the promising role of mannose-based interfaces in mimicking natural *E. coli*–carbohydrate interactions, achieving high selectivity, superior sensitivity, and stability. However, their reliance on FimH-mediated binding may limit detection to specific strains, and performance in complex real-world samples remains uncertain.

**Fig. 2:**
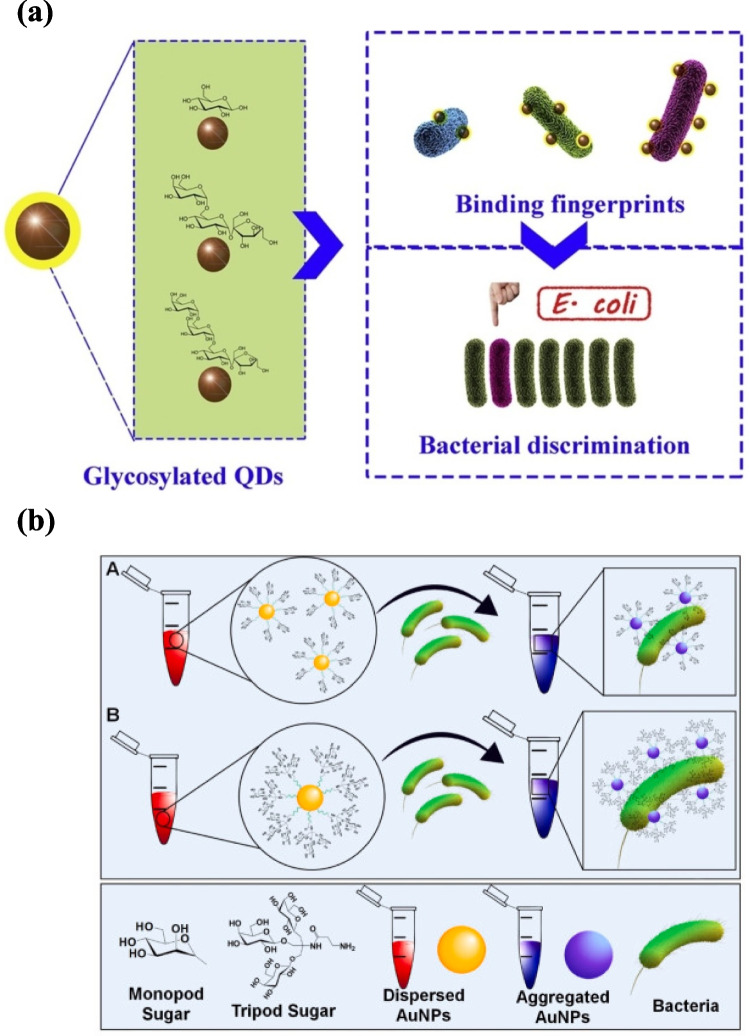
Glycan-functionalized nanomaterials for rapid bacterial detection and discrimination: **a** multivalent glycosylated Cu:CdS quantum dots [58]; **b** AuNPs functionalized with monopod (A) and tripod (B) glycans [59]. Reproduced with permission from Elsevier

Many versatile multivalent colorimetric biosensors have been developed based on carbohydrate-functionalized nanoparticles, including Au, Cu, CdS quantum dots, and conjugated polymers. These glyconanomaterials offer impressive speed and multiplexing capability; however, their use is limited by synthetic complexity, quantum dot toxicity, and absent standard protocols. For instance, Qi et al. introduced multivalent glycosylated Cu:CdS quantum dots (QDs) with strong fluorescent properties. Three natural carbohydrates, glucose, stachyose, and raffinose, were conjugated to the QDs to mimic natural multivalent cellular interactions, enabling rapid discrimination of six bacterial strains (*P. aeruginosa*, *M. luteus*, *E. coli*, *V. alginolyticus*, *S. algae*, and *D. desulfuricans*) via linear discriminant analysis (LDA), achieving 91.6% classification accuracy within 30 min (Fig. 2a) [57]. Yet, the study lacks a detailed comparative framework—such as varying glycan types or linker architectures—which limits its generalizability. Priyadarshi et al. present mannose- and galactose-functionalized nanoglycoclusters employing AuNPs and PEG linkers, differentiated lectins, and bacteria via monopod and tripod glycans. Mannose monopod/tripod (MM, MT) targeted *E. coli* and concanavalin A, while galactose monopod/tripod (GM, GT) bound *Pseudomonas aeruginosa* and peanut agglutinin lectin. Tripod glycans exhibited higher affinity than monopods (Fig. 2b). Their comparative approach is commendable for its mechanistic insight, demonstrating how glycan architecture governs sensitivity, while glycan diversity modulates specificity [58].

**Fig. 3:**
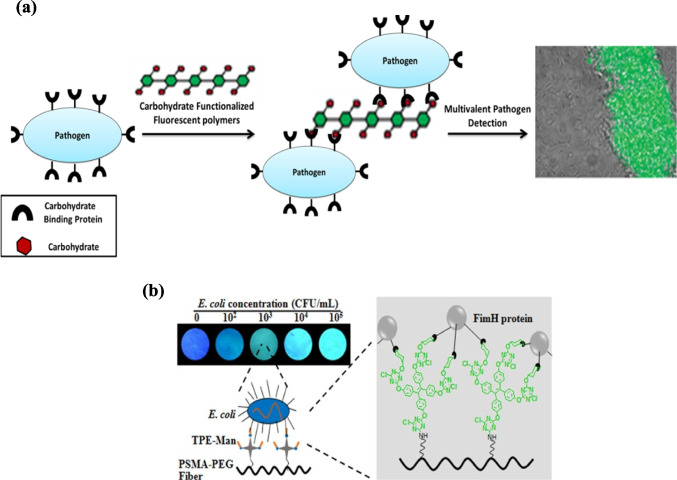
Fluorescent carbohydrate-based probes for *E. coli* detection: **a** mannose-functionalized polymers (PPEs) [61]; **b** mannose-functionalized TPE fibrous mats under 365 nm UV light [62]. Reproduced with permission from ACS

Expanding beyond QDs, several carbohydrate-functionalized conjugated polymers have been explored through visual and spectroscopic signals. Water-soluble mannose-functionalized glycopolythiophenes enabled visible absorption spectroscopy, where carbohydrate incorporation perturbs the twisted conformation of the polymer backbone, and binding of specific* E. coli* lectins induced characteristic red shifts [34]. Similarly, Ma et al. reported a mannose-modified polydiacetylene (PDA) to fabricate a Langmuir–Blodgett (LB) film that selectively recognized *E. coli*, producing visually distinct, detectable color changes confirmed spectroscopically [59]. Although reliance on visual colorimetric readouts may limit quantitative precision, Disney et al. designed multivalent mannose-functionalized fluorescent poly(p-phenylene ethynylene; PPE) polymers that enabled both visualization and quantification of *E. coli*, yielding fluorescent cell clusters within 10–15 min and achieving a detection limit of 10^4^ cells (Fig. 3a) [60]. Zhao et al. further developed rapid test strips based on electrospun polystyrene-co-maleic anhydride (PSMA) fibers conjugated with mannose and tetraphenylethylene (TPE). These fibers bind selectively to FimH proteins of *E. coli* fimbriae, producing a TPE “turn-on” signal via aggregation-induced emission (AIE) that enabled visual identification of bacteria with loads as low as 10^2^ CFU/mL within minutes, and a linear response from 10^2^ to 10^5^ CFU/mL (Fig. 3b) [61]. Future work should emphasize enhancing robustness in complex matrices, expanding strain coverage, and validating these systems under diverse conditions, while addressing polymer stability and scalability.

**Fig. 4:**
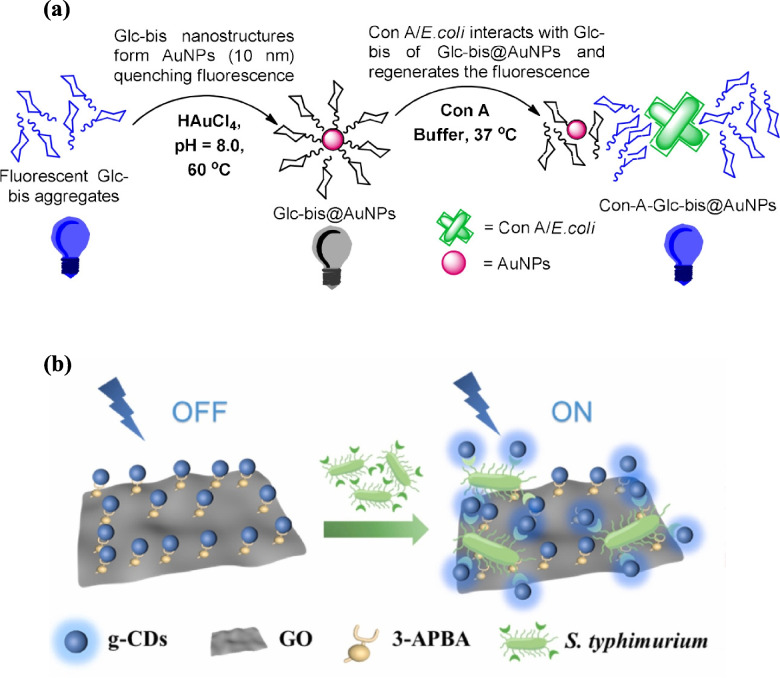
Fluorescence-recoverable biosensors: **a** Glc-bis@AuNPs with quenched fluorescence restored upon interaction with Con A or *E. coli* [64]. Reproduced with permission from ACS; **b** glycopeptide-mimetic carbon dots (g-CDs) with graphene oxide (GO) and 3-aminophenylboronic acid (3-APBA) for *S. typhimurium* detection [65]. Reproduced with permission from Elsevier

Similarly, the AIE properties of glucose-based monoacrylamide and bisacrylamide were investigated under physiological conditions, demonstrating applications in cell imaging and the detection of Concanavalin A (Con A) and *E. coli*. These fluorescent glycopolymer probes exhibited high water solubility, photostability, and multivalent binding, with fluorescence intensity showing a linear correlation with *E. coli* concentrations from 1.0 × 10⁶ to 1.7 × 10⁸ CFU/mL and a detection limit of 7.3 × 10^5^ CFU/mL within ~ 20 min [62]. In parallel, glucose-based bis(acrylamide) (Glc-bis) stabilized gold nanoparticles (AuNPs) acted as a turn-on fluorescence sensor, where emission quenched by AuNPs was restored upon binding with Concanavalin A (Con A), showing a detection limit of 1.6 nM. Inspired by this, Glc-bis@AuNPs were tested with *E. coli* strains: FimH-positive K12 (BW25113) and FimH-negative TOP10. The sensor showed 1.5- and 2-fold emission increases at 10^4^ and 10⁷ CFU/mL K12, respectively, but no significant change with TOP10 (Fig. 4a) [63]. Although carbohydrate-based AIE systems show promise for rapid pathogen detection, their high detection thresholds limit clinical utility, highlighting the need for enhanced sensitivity and rigorous validation.

##### Salmonella

*Salmonella*, a Gram-negative enterobacterium with *S. typhimurium* as the most common cause of salmonellosis, adheres to host cells via fimbrial interactions with mannose-containing glycoproteins. A fluorescent-recoverable sensor exploiting this mechanism employed glycopeptide-mimetic carbon dots (g-CDs) derived from mannose and histidine with graphene oxide–phenylboronic acid (GO-PBA) as a quencher, achieving a detection limit of 2370 CFU/mL, a linear range of 10^4^–10⁷ CFU/mL, and 93–103% recovery in chicken samples (Fig. 4b) [64]. Complementarily, a carbohydrate-functionalized dynamic double emulsion sensor targeting *Salmonella enterica* utilized reversible boronic acid–carbohydrate interactions, with B(OH)₂₁-mannan droplets detecting < 10^4^ CFU/mL within 2 h; antibody functionalization enhanced selectivity, though further optimization is needed to lower detection thresholds and confirm robustness in complex food matrices [65]. Both introduce innovative fluorescence-based strategies for *Salmonella* detection using carbohydrate-specific interactions, yet each has limitations. The double-emulsion sensor offers high responsiveness, but its complex droplet fabrication may hinder scalability, while the g-CDs/GO-PBA probe provides better practicality and stability, though its detection limit remains moderate.

##### ***Helicobacter pylori***

*H. pylori* persistently infects the stomach lining of billions of individuals across the globe. Colonization is initiated by adhesion to sialylated and sulfated oligosaccharides in salivary and gastric mucins. An optical biosensor simulating gastric conditions was developed using a cuvette-based resonant mirror platform, where human gastric mucin fractions revealed with diverse terminal carbohydrate structures enabled detailed analysis of *H. pylori* interactions and improved performance over conventional assays [66]. Likewise, BabA is the best-characterized *H. pylori* adhesin, exhibiting high specificity for the Lewis b (Leb) blood group antigen, a fucosylated carbohydrate expressed on gastric epithelial cells and in saliva. This interaction has been exploited in surface plasmon resonance (SPR) and double resonance long-period grating (DR LPG) biosensors. These label-free platforms achieved sensitivities of 3000 CFU/mL (SPR) and 10^2 ^CFU/mL (DR LPG), surpassing the rapid urease test and highlighting their potential for non-invasive *H. pylori* detection [67]. However, the approach’s reliance on a single adhesin–receptor pair may limit its applicability across diverse *H. pylori* strains with variable BabA expression.

#### Toxins

##### Cholera toxin

Cholera toxin (Ctx), an AB5 toxin produced by *Vibrio cholerae*, binds with high specificity to GM1 gangliosides on host cells, making this interaction a valuable basis for carbohydrate biosensor design [32]. A promising bioinspired electrochemical platform, such as GM1-expressing Caco-2 cell membrane (CCM) attached to an electrode surface via the vesicle fusion method, is proposed. This CCM-coated biosensor closely mimics the natural binding and achieves detection limits of ~ 11.46 nM with a range of 100 ng/mL to 1 mg/mL by impedance spectroscopy. However, its dependence on cell-derived membranes may hinder reproducibility, scalability, and cost-effectiveness for widespread use [68]. In contrast, colorimetric approaches using GM1–poly(diacetylene) liposomes-based artificial membranes enabled rapid naked-eye detection within 2 min, though they offer limited quantification [69]. Similarly, lactose-stabilized gold nanoparticles that mimic the GM1 ganglioside pentasaccharide show naked-eye color change within 10 min, with a detection limit of 54 nM (3 µg/mL), balancing simplicity with moderate sensitivity [70].

More sensitive strategies, a flow cytometry-based biosensor exploiting multivalent GM1-functionalized phospholipid bilayers using fluorescence resonant energy transfer (FRET) readouts, enabled CTx detection (10 pM, 30 min) [71]. The same group advanced a two-tiered FRET strategy by tagging donor, acceptor, and intermediate fluorophores on receptors, causing significant reduction in background fluorescence and enhanced sensitivity and selectivity, achieving CT detection down to 10 pM [72]. However, the complexity of probe design and covalent tagging may limit scalability and reproducibility. Added validation in biologically relevant samples is needed.

##### Shiga toxin (Stx)

Shiga toxin (Stx), produced by *Shigella dysenteriae* type 1 and Shiga toxin–producing *E. coli* (STEC), is an AB5 cytotoxin whose B subunit binds specifically to the globotriaosylceramide (Gb3/Pk trisaccharide) receptor, a key step in cytotoxicity [73]. Miura et al. reported a QCM biosensor using self-assembled monolayers (SAMs) of Gb3 mimics “glyco-cluster” with different alkyl chain lengths to study the molecular basis of Shiga toxin recognition and binding mechanisms. The monolayer configuration enables precise control of receptor density and orientation. Results show distinct binding preferences: Stx1 favoring short-chain ligands and Stx2 preferring longer alkyl chains [74].

Colorimetric approaches using Gal-α1,4-Gal glycopolydiacetylene (GPDA) nanoparticles enabled rapid, selective, and quantitative detection of STEC with a detection limit of 1200 units/µL, highlighting its potential for routine monitoring of STEC in food and water [75]. Multivalent globotriose (Pk ligand)-functionalized gold nanoparticles (Pk-AuNPs), which mimic the natural clustering of carbohydrate ligands on cell surfaces, were synthesized in six variants differing in size and linker length. SPR assays showed that affinity depended on nanoparticle size, linker length, and ligand density. A 20-nm Pk-AuNP with a long linker exhibited a > 10⁸-fold higher affinity than monovalent Pk, enabling efficient toxin capture from crude lysates [76]. Building on this, a synthetic glycopeptide modified with globotriose, mimicking the natural toxin receptor, was enzymatically synthesized and immobilized on AuNPs coated with a porphyrin-based polymer (Au@FePor-TFPA-COP). Incorporation of a modular peptide linker enhances glycan orientation and accessibility, improving binding efficiency and yielding a visible blue color at nanomolar concentrations within 30 min. This biosensor offers high sensitivity, specificity, and stability, enabling rapid Shiga toxin detection [77]. Additionally, Gb3-functionalized silicon nitride (SiN) chips were employed for the detection of Stx1 using reflectometric interference spectroscopy (RIfS). Gb3 immobilization was optimized through two strategies—click chemistry and NHS coupling—with click chemistry exhibiting superior performance. Incorporation of a triethylene glycol spacer enhanced orientation control and reduced steric hindrance, improving ligand accessibility. The sensor achieved a detection limit as low as 100 ng/mL with minimal cross-reactivity to non-specific lectins and was successfully applied to cucumber samples, highlighting its potential for food safety monitoring. Additionally, the chip could detect Ricinus communis proteins, highlighting its multiplexing capability [78].

Together, these platforms emphasize how multivalency, ligand presentation, and surface chemistry govern the performance of Stx diagnostics, revealing inherent trade-offs between sensitivity, simplicity, and integration in glycan-based biosensing

#### Virus

##### SARS-CoV-2

**Fig. 5:**
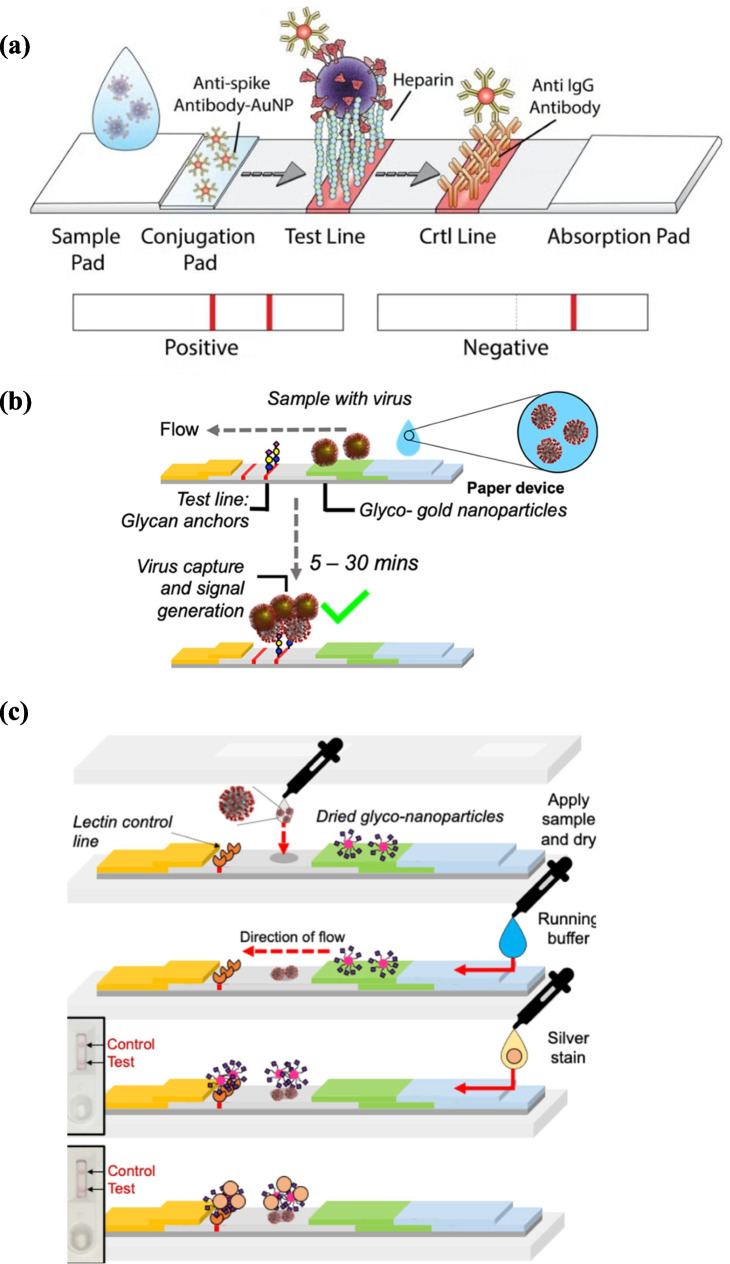
Glycan-based lateral flow (LF) assays for SARS-CoV-2 detection: **a** schematic of the GlycoGrip LF biosensor, where the sample migrates along the strip, viral particles bind to conjugated antibodies, and are captured at the glycopolymer test line [82]; **b** multivalent N-acetylneuraminic acid–PHEA assembled on citrate-stabilized AuNPs immobilized within the strip [80]; **c** device optimization employing the sample itself as the test line [83]. Reproduced with permission from ACS

**Fig. 6:**
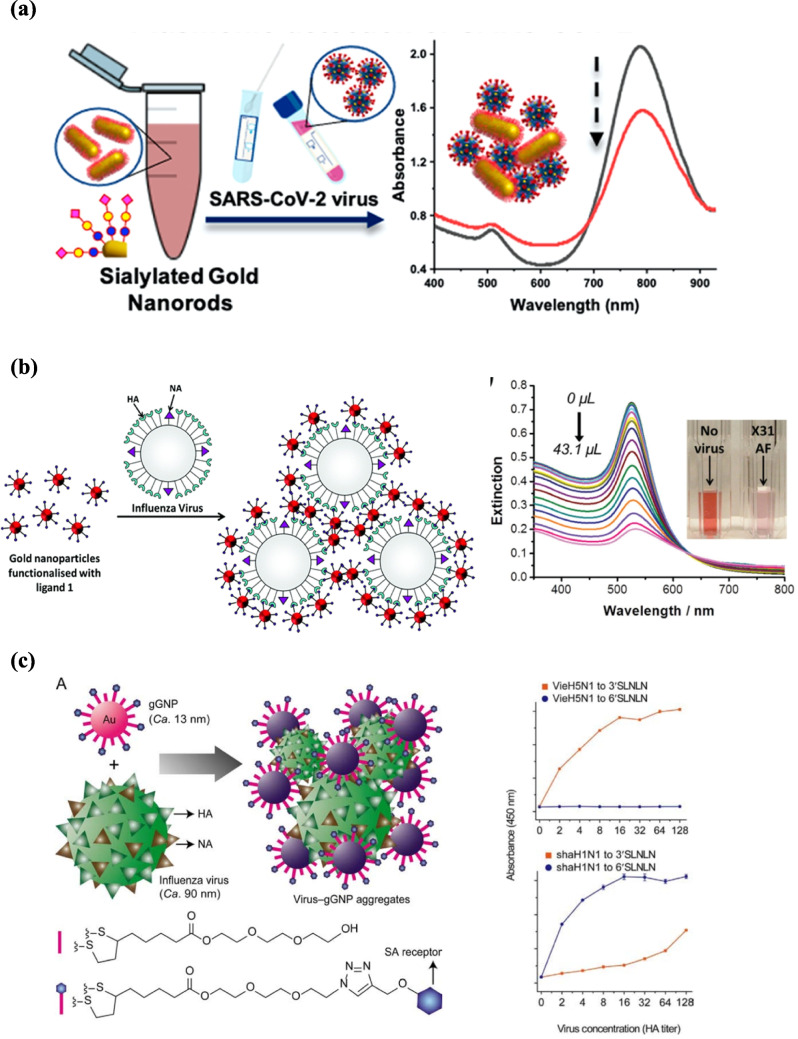
Glycan-functionalized gold nanoparticles (gGNPs) for viral detection: **a** glycosylated nanorod binding the SARS-CoV-2 spike protein, showing dose-dependent responses [84]. Reproduced from ACS; **b** gGNP aggregation upon interaction with influenza HA, with corresponding UV–Vis spectra [88]. Reproduced from RSC; **c** influenza detection via SA–virus binding–induced gGNP aggregation, producing a visible color change quantified by UV–Vis spectroscopy with glycan-binding properties of vieH5N1 and shaH1N1 analyzed by glycan-based ELISA. Reproduced with permission from Elsevier [89]

Significant advancements in carbohydrates-based sensors for virus detection have focused on SARS-CoV-2, the coronavirus responsible for the COVID-19 pandemic. Sialic acids, key receptors for the viral spike S1 subunit, mediate host cell attachment and have been exploited to develop diagnostic devices that use glycans instead of antibodies as recognition elements [79]. To meet the urgent demand for rapid virus detection, many lateral flow assays have been developed. Zhongping and colleagues designed heparan sulfate (HS) polysaccharide microarrays and applied SPR to study coronavirus S proteins (MERS-CoV, SARS-CoV-2, and SARS-CoV) binding, revealing that all tested proteins bind to HS. This study provides the biochemical foundation for glycan-based biosensor development [80]. As a consequence, Ronit Freeman and colleagues developed GlycoGrip, a universal lateral flow sensor for SARS-CoV-2 and its variants. The assay used heparan sulfate (15 kDa) and heparin polymers for viral capture and anti-spike antibody–gold nanoparticles for signal generation (Fig. 5a). GlycoGrip sensitively distinguished SARS-CoV-2 variants in bodily fluids, detecting spike protein at concentrations as low as 3.13 µg/mL (range 3.13–50 µg/mL), with results visible to the naked eye [81]. A glycan-based lateral-flow assay has been developed using multivalent N-acetylneuraminic acid–functionalized PHEA-coated gold nanoparticles as both capture and detection agents, targeting the sialic acid–binding site of the SARS-CoV-2 spike protein (Fig. 5b). The assay provided results within 30 min with a detection limit of ~ 5 µg/mL for spike protein and 1.5 × 10^4^ units/mL for a pseudotyped lentivirus presenting spike protein, demonstrating proof-of-principle for glyco-lateral flow devices in outbreak monitoring [79]. Further optimization led to a flow-through format, where the sample itself served as the test line (Fig. 5c), supported by a polymeric coating that minimized nonspecific binding and enabled glycan presentation. This platform specifically recognized spike-bearing lentiviruses and recombinant spike protein, even after heat or detergent treatment, and achieved 85% sensitivity and 93% specificity with RT-PCR–validated swab samples. The prototype successfully detected virus in heat-inactivated patient swabs, also detected mutant spike proteins, suggesting robustness against variants [82]. This represents the first all-glycan lateral flow system for point-of-care diagnostics. The same group developed plasmonic glycosylated polymer-stabilized gold nanorods functionalized with 2,3-sialyllactose for SARS-CoV-2 detection in clinical samples. These nanorods showed dose-dependent responses to recombinant spike protein, generating a detectable signal at 40 µg/mL, and the results were validated with patient swab samples (Fig. 6a) [83]. As a whole, they underscore the versatility of glycan-based recognition—from understanding viral tropism to engineering rapid, scalable diagnostics that could be extended to other pathogens and provides a complementary alternative to antibody-based testing.

##### Influenza virus

The influenza virus, known for its high mutation rate and host adaptability, drives both seasonal outbreaks and major pandemics, underscoring the need for continuous monitoring [84]. Infection begins when viral hemagglutinin (HA) binds to host cell glycans terminating in sialic acid (SA). The type of SA linkage determines host specificity: human-adapted strains prefer α2,6-linked SA (NeuAcα2,6Gal) found in the upper respiratory tract [85], while avian and swine strains favor α2,3-linked SA in the intestinal mucosa. These differences in glycan structures have inspired the development of glycan-based biosensors for selective detection of influenza viruses and strain differentiation [33].

Colorimetric glycan sensors represent one of the earliest approaches. Charych et al. pioneered a polydiacetylene (PDA) liposome expressing sialic acid residues mimicking the spatial organization of cell membranes. The sensor undergoes a visible color change (blue/purple to pink/orange) upon HA–SA binding, enabling detection down to 11 HAUs (~ 1.1 × 10⁷ particles) with specificity confirmed by competitive inhibition [86]. Building on this principle, Baek et al. developed water-soluble glycopolythiophenes functionalized with sialic acid ligands for influenza virus detection via visible absorption spectrometry. Virus binding induced a backbone transition from nonplanar to planar, providing a quantifiable optical signal, a red shift ranging from 0 to + 27 nm for influenza A and 0 to + 32 nm for influenza B [34]. Critically, while both systems leverage glycan–virus interactions, the PDA-based sensor excels in visual simplicity and membrane mimicry, whereas the glycopolythiophene platform offers enhanced spectral resolution and solubility. These early innovations laid the groundwork for more sophisticated glycan-based biosensors, highlighting the utility of conformationally responsive polymers in virus diagnostics.

Gold nanoparticle (GNP)–based platforms have further advanced this concept, demonstrating strong potential for rapid and selective influenza detection. Marín et al. developed a plasmonic bioassay using GNPs functionalized with a trivalent α2,6-thio-linked sialic acid derivative. Virus binding triggered a visible color change within 30 min, and the system successfully distinguished human influenza (α2,6-binding) from avian influenza H5N1 (α2,3-binding), demonstrating high specificity (Fig. 6b) [87]. This study demonstrated the feasibility of using glycan topology as a molecular barcode for viral typing. Building on this concept, Zheng et al. developed a more refined system using both α2,3 and α2,6 sialylated glycan-functionalized GNPs (gGNPs) for a single-step colorimetric assay (Fig. 6c). Virus-induced gGNP aggregation produced a red-to-purple shift measurable by spectrophotometry, enabling the differentiation of 14 influenza strains—including major human and avian subtypes—from each other and from respiratory syncytial virus [88]. Together, these GNP-based strategies highlight the promise of simple, portable sensors for influenza diagnosis and surveillance.

More sophisticated biosensing strategies leverage microarrays and nanodevices. Zhang et al. constructed a carbohydrate-based receptor analogue microarray biosensor using the arrayed imaging reflectometry (AIR) platform. The microarrays, featuring immobilized α2,6- and α2,3-linked glycans analogues, enabled label-free multiplex detection of glycan-binding lectins. Specifically, *Sambucus nigra* agglutinin (SNA) and *Maackia amurensis* agglutinin (MAA) were used to selectively bind α2,6- and α2,3-linked glycans, respectively. This system successfully differentiated between human influenza A/California/07/2009 (H1N1pdm) and avian influenza A/Netherlands/1/2000 (H13N8). The microarrays showed a concentration-dependent response consistent with Langmuir binding behavior, with a detection limit of 0.06 µg/mL for lectins and 9.29 × 10^5^ pfu/mL for H1N1pdm. This approach could be expanded to include a broader range of glycans and virus subtypes, offering a valuable tool for monitoring influenza adaptation and supporting global surveillance efforts [24].

**Fig. 7:**
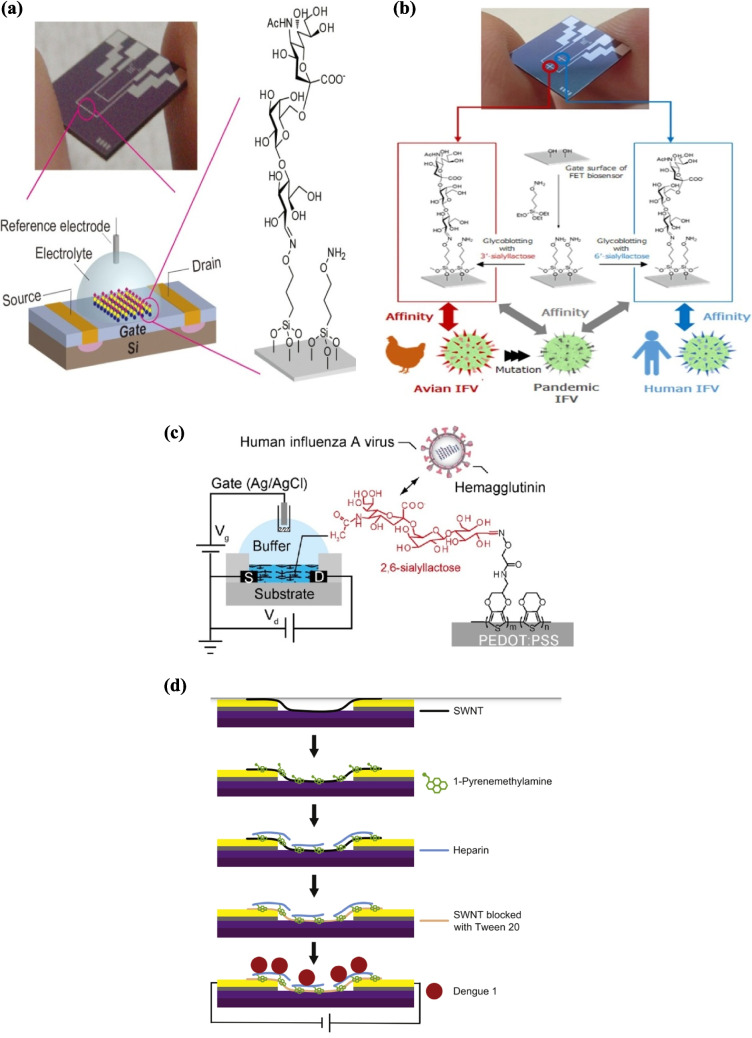
Glycan-functionalized electronic biosensors for viral detection: **a** field-effect transistor (FET) sensor with carbohydrate biointerfaces based on self-assembled monolayers (SAMs) [90]. Reproduced with permission from ACS; **b** dual-channel FET biosensor for pandemic influenza particle detection via glycan immobilization [91]. Reproduced from Springer Nature; **c** organic electrochemical transistor (OECT) for influenza detection using glycan-modified sensing surfaces (PEDOT:PSS: poly(3,4-ethylenedioxythiophene):poly(styrenesulfonate)) [93]. Reproduced from Elsevier; **d** chemiresistor fabricated with single-walled nanotubes (SWNTs) for dengue virus detection [95]. Reproduced from Elsevier

FET (field-effect transistor)–based glycan biosensors have been applied for ultrasensitive influenza detection. Sho et al. constructed a silicon nanowire FET sensor functionalized with trisaccharide receptors terminating in either α2,6- or α2,3-linked sialic acids (Fig. 7a). The device detected as few as 60 H5N1 or 6000 H1N1 hemagglutinin proteins in a 20 µL sample—equivalent to a single H5N1 or 12 H1N1 virus particles—with a dynamic range covering nine orders of magnitude (from attomolar to nanomolar concentrations) [89]. The group further developed a dual-channel label-free FET system modified with 6′- and 3′-sialyllactose on separate gates, enabling direct and specific detection of H1N1 and H5N1 particles in nasal mucus (Fig. 7b). This platform showed a detection limit of 10^0.5^ TCID_50_/mL and a working range from 10^0.5^ to 10^8.5^ TCID_50_/mL [90], highlighting its potential for early influenza diagnosis and outbreak monitoring.

Electrochemical and mass-sensitive biosensors have also demonstrated significant potential for viral detection. Hushegyi et al. reported an ultrasensitive impedimetric biosensor with 2,3-sialyllactose immobilized on polycrystalline gold electrodes, achieving attomolar sensitivity for hemagglutinin, rivaling advanced FET-based platforms [91]. Hai et al. further developed an organic electrochemical transistor (OECT) incorporating a PEDOT channel functionalized with 2,6-sialyllactose, where virus binding induced drain current changes, enabling sensitive detection in aqueous media. The OECT’s low power consumption and printability highlight its promise for real-time, portable influenza monitoring (Fig. 7c) [92]. Complementing these systems, Horiguchi et al. designed a QCM biosensor functionalized with 6′-sialyllactose, achieving rapid, label-free detection of H1N1 within minutes, with superior sensitivity to conventional immunochromatographic assays—detecting as little as 1/16 HAU in 10 min and 1/80 HAU in 30 min, independent of viral strain variability [93].

##### Dengue virus

Dengue virus (DENV), a Flaviviridae member, is a major public health threat in tropical and subtropical regions. Infection ranges from asymptomatic cases to dengue fever, dengue hemorrhagic fever, and the potentially fatal dengue shock syndrome [94]. During infection, the virus exploits host receptors such as heparin, a structural analog of heparan sulfate proteoglycans, which facilitates viral entry into Vero cells and liver cells (hepatocytes). Building on this interaction, Wasik et al. developed a chemiresistive biosensor for whole dengue virus detection using a single-walled carbon nanotube (SWNT) network functionalized with heparin oligosaccharides (Fig. 7d). The SWNT network was self-assembled on interdigitated gold electrodes, with heparin attached via cross-linking through a pyrene linker (1-pyrenemethylamine). The sensor successfully detected dengue virus type 1 in both phosphate buffer and cell culture, showing high selectivity, with influenza H1N1 serving as a negative control [95].

## Conclusions and future directions

Carbohydrate-based biosensors represent a rapidly advancing and highly promising frontier in pathogen detection, integrating the inherent specificity of glycan–protein interactions with diverse transduction platforms. Their ability to mimic natural glycan presentations, through multivalent and structurally engineered interfaces, enables precise recognition of microbial lectins and adhesins, offering a robust bioinspired alternative to antibody- or aptamer-based systems. Advances in carbohydrate-functionalized nanomaterials, polymers, and hybrid membranes have led to remarkable improvements in biosensing performance, facilitating the detection of clinically relevant pathogens. Despite these achievements, several barriers continue to limit their translation into routine diagnostic use. Major challenges include cross-reactivity and variability in glycan density and orientation and difficulties in the reproducible synthesis or isolation of well-defined carbohydrate ligands. Moreover, while highly biomimetic architectures such as cell membrane–coated electrodes or mucin-inspired platforms enhance physiological relevance, they often suffer from high cost and limited scalability. In contrast, synthetic glycan analogues, though simpler and more reproducible, may inadequately capture the complexities of natural host–pathogen recognition.

Future development should therefore focus on integrating advanced glycoengineering, micro/nanofabrication, and computational modeling to rationally design stable, multivalent, and structurally controlled glycan interfaces to preserve functionality in complex biological matrices. Combining these with miniaturized and multiplexed detection systems could yield portable, cost-effective, and high-throughput next-generation diagnostic devices capable of real-time pathogen surveillance. Ultimately, carbohydrate-based biosensors hold the potential to bridge the gap between fundamental glycobiology and practical point-of-care diagnostics, providing a powerful platform for early infection detection, biosafety monitoring, and global health applications.
